# Associations of cognitive impairment with self-isolation and access to health and care during the COVID-19 pandemic in England

**DOI:** 10.1038/s41598-023-31241-3

**Published:** 2023-03-28

**Authors:** Brian Beach, Nicholas Steel, Andrew Steptoe, Paola Zaninotto

**Affiliations:** 1 UCL Research Department of Epidemiology & Public Health, University College London, London, UK; 2 Norwich Medical School, University of East Anglia, Norwich, UK; 3 UCL Research Department of Behavioural Science & Health, University College London, London, UK

**Keywords:** Epidemiology, Dementia, Health services

## Abstract

This research explored experiences across three cognitive function groups (no impairment, mild impairment, and dementia) with respect to shielding (either self-isolating or staying at home), COVID-19 infection, and access to health/care services during the COVID-19 pandemic. Analyses were conducted using data from the English Longitudinal Study of Ageing (ELSA) COVID-19 sub-study collected in 2020. We report bivariate estimates across our outcomes of interest by cognitive function group along with multivariate regression results adjusting for demographic, socioeconomic, geographic, and health characteristics. Rates of shielding were high across all cognitive function groups and three measured time points (April, June/July, and Nov/Dec 2020), ranging from 74.6% (95% confidence interval 72.9–76.2) for no impairment in Nov/Dec to 96.7% (92.0–98.7) for dementia in April (bivariate analysis). 44.1% (33.5–55.3) of those with dementia experienced disruption in access to community health services by June/July compared to 34.9% (33.2–36.7) for no impairment. A higher proportion of those with mild impairment reported hospital-based cancellations in June/July (23.1% (20.1–26.4)) and Nov/Dec (16.3% (13.4–19.7)) than those with no impairment (18.0% (16.6–19.4) and 11.7% (10.6–12.9)). Multivariate adjusted models found that those with dementia were 2.4 (1.1–5.0) times more likely than those with no impairment to be shielding in June/July. All other multivariate analyses found no statistically significant differences between cognitive function groups. People with dementia were more likely than people with no impairment to be shielding early in the pandemic, but importantly they were no more likely to experience disruption to services or hospital treatment.

## Introduction

The number of people living with cognitive impairment is expected to grow in coming decades, which will place increasing pressures on health and care services as well as the general population through informal caregiving and economic costs^1^. Successfully adapting to these changes requires a better understanding of the social and health impacts that people with cognitive impairment face, along with the inequalities that shape their experiences.

Moreover, the COVID-19 pandemic has presented new challenges for individuals and governments alike. Although COVID-19 emerged recently, the research community has actively adapted in collecting data and reporting on findings throughout the pandemic. As a result, some findings and insights have emerged since the start of the pandemic related to the experiences of people with cognitive impairment.

Older people with dementia, for example, are highly vulnerable, relying on caregivers for day-to-day activities^2^; they are therefore likely to be disproportionately affected by lockdown, self-isolation, and social distancing^3,4^. When their normal routines are disturbed, they may be more susceptible to rapid declines in health and wellbeing resulting from social isolation^5,6^. In the United Kingdom (UK), people with dementia have been identified as the hardest hit by the pandemic^7^.

People with cognitive impairment may have difficulty understanding the social changes around them resulting from lockdowns. Similarly, they may find it challenging to comprehend public health messages around the kinds of behavioural adaptations required during the pandemic. Current evidence on this is somewhat mixed. One study in Germany found that 85% of people (all with cognitive impairment) were sufficiently informed about COVID-19 measures, while 64% thought such measures were appropriate^8^. Social activities like going out and meeting others were also pursued less frequently. However, another study found that compliance with new measures was one area most affected among older Greek people with mild cognitive impairment and dementia^9^.

Studies in Japan also found that people with dementia or mild cognitive impairment were less likely to understand the situation related to the pandemic and to comply with restrictions around social distancing and the use of face masks^10–13^. The degree of compliance for people with cognitive impairment may be shaped by whether they live alone or with other family, as those living alone did not change their frequency of going out while those living with family did^10^.

If people with cognitive impairment were less likely to follow the protective guidelines related to COVID-19, they may have put themselves at higher risk of infection. Such higher risk of infection has been reported in the UK and other countries including the United States^14–16^. The pandemic has also impacted every aspect of healthcare, including the postponement of planned treatment and a higher disinclination among people to pursue screening and treatment for non-COVID-19 condition^17,18^. As people with dementia are at greater risk of adverse outcomes from other conditions compared to those of similar age without dementia^19,20^, such shifts in health-related activity may lead to excess morbidity and mortality through and after the pandemic.

Compliance with pandemic-related guidance plays a key part in keeping people healthy and well, and avoiding adverse outcomes depends on obtaining necessary health and care services. Discontinued care can raise concerns around faster cognitive decline and has been linked to increased caregiver burden in the UK and the Netherlands^21,22^. Lockdown also had a strong impact on the provision and utilisation of health care services such as day clinics, relief services, and prescribed therapies in Germany and the UK^8,23^. Another UK study found that people with dementia and their caregivers avoided contact with primary care services^24^. The pandemic also negatively impacted ability of people with dementia to engage in self-care and manage everyday activity^25^.

A clear picture on the impact of the pandemic for people with cognitive impairment is crucial for policymakers and health services to adequately plan for future demands^26^. This paper investigates the experiences of people with differing degrees of cognitive function in England to shed light on some short-term impacts of the pandemic on them. The study examined the experiences among people with cognitive impairment compared to those without with respect to shielding (either self-isolating or staying at home), COVID-19 infection, access to community health and care services, and hospital-based treatments.

## Methods

### Data

Our project used data collected before and throughout the COVID-19 pandemic as part of the English Longitudinal Study of Ageing (ELSA)^27^. ELSA follows a representative sample of people aged 50+ across England since 2002, asking about topics including health, wellbeing, finances, and social connections.

Critical to this analysis was the COVID-19 sub-study conducted as part of ELSA in 2020^28^. A direct and rapid response to the pandemic, ELSA members and their partners provided responses to two special surveys conducted in June/July and November/December 2020, capturing their perspectives during the pandemic. Retrospective data were also collected, e.g. about experiences in April 2020, providing three time points of data for most respondents on some key questions.

In prior waves of ELSA, responses were collected via computer-assisted personal interviews (CAPI). In contrast, the COVID-19 sub-study was administered through a mix of internet- and phone-based questionnaires. Also different to the main ELSA interviews, the COVID-19 sub-study did not allow proxy interviews, although people who needed assistance with the internet-based surveys were allowed to seek help from other people.

The cognitive function groups were derived from a series of objectively administered tests and cognitive batteries administered pre-COVID, as described in the next sub-section. The cognitive function module was not administered during the COVID sub-study, but new dementia diagnoses were included. Since the use of self-reported measures may be impacted by an individual’s cognitive status, the interpretation of results is underpinned by the understanding that responses (e.g. about health status) reflect the individual’s perception rather than any objective measure. For those with more advanced cognitive impairment, this may introduce bias.

Response rates for the COVID-19 sub-study were notably high, at 75% for each wave of data collection and 94% longitudinally. The representativeness of ELSA over time has been maintained by the inclusion of refreshment samples at multiple waves, and representativeness in this study was facilitated by the inclusion of non-response sampling weights. Further details on the survey design, methodology, and performance of the ELSA surveys can be found in the technical reports and other study documentation^27–30^.

### Measures

Our outcomes of interest relate to shielding, probable infection with SARS-CoV-2, disruption in access to health and care services, and cancellations of hospital operations or treatments.

Shielding combines responses asking if respondents were self-isolating or staying at home; staying at home differed from self-isolating in allowing for short ventures out of the home for necessary shopping, medical care, or exercise. Respondents were asked about this with respect to the week prior to the interview at both waves (giving results for June/July and for Nov/Dec) and in April; April results are based on responses at the June/July wave. Our measure does not reflect the clinical concept related to vulnerable people taking self-protective action, nor does it imply any evaluation of whether respondents received specific advice to shield from medical authorities at the start of the pandemic.

For probable infection, in the first wave of the sub-study, respondents were asked about particular symptoms of illness they had experienced. We used the three key symptoms associated with COVID-19—a high temperature, a new continuous cough, and a loss of the sense of smell or taste—to create a measure of probable infection with SARS-CoV-2. Reports of any of these symptoms were combined with reports of a positive diagnosis or hospitalisation due to COVID-19 to assess probable infection.

With respect to disruption in community health and care services, respondents were asked at the first wave if they had been able to access the services they need since the outbreak of the pandemic; examples included a dentist, podiatrist, counselling, or personal care. The question in Nov/Dec explicitly mentioned the general practitioner (GP), while access to the GP was part of a separate question in June/July; these responses have been combined for June/July. At the second wave, those present at the first wave were asked about their experience since the previous interview, while new respondents were asked about experiences since the outbreak in February (in the UK). In addition to yes and no, response options included “did not attempt to contact” or “did not need to contact”. For our analyses, we have considered “did not attempt to contact” as implying need.

Questions about cancellations of hospital-based operations and treatments were asked at both waves of the sub-study, with slight differences in response options. Both waves allowed yes or no responses, while the second wave also included the option, “I did not have a hospital operation or treatment booked.”

The main exposure in our analysis is cognitive function status, classified as no cognitive impairment, mild cognitive impairment, or dementia. Individuals were classified into one of these groups using work from another ELSA sub-study from 2018, the Harmonised Cognitive Assessment Protocol (HCAP). ELSA-HCAP took a nuanced look at participants’ cognitive function, applying a range of questionnaires and other evaluations used to assess cognition in clinical and non-clinical settings^29^. From this work, a predictive algorithm was developed to classify all ELSA respondents into one of three cognitive function groups: no impairment, mild impairment, or dementia^30^.

### Analytical approach

To assess the association between cognitive function and our outcomes of interest, we applied binomial logistic regression, adjusting for key confounders and reporting odds ratios (ORs). A multinomial logistic model was used for hospital-based cancellations in Nov/Dec to account for the variance among those with no treatments booked, reported using relative risk ratios (RRRs). All models controlled for demographics (age, gender, ethnicity, co-habiting partnership status), socioeconomics (education, wealth, and employment status), geography (urban/rural and English region), and health (self-rated health and the presence of multimorbidity). Analyses were adjusted to account for sample design (clustering and stratification) and non-response bias (using cross-sectional weights). Our full analytical sample included 5116 respondents for June/July and 4904 for Nov/Dec, with 4737 respondents present at both waves.

### Ethical approval

Ethics approval is not applicable for this study. The study made exclusive use of secondary data analysis.

## Results

We present descriptive statistics for the sample across our control variables according to cognitive function status in Table 1, reporting the complete-case analytical sample from the June/July ELSA COVID-19 data.

**Table 1:** Descriptive statistics for the June/July ELSA COVID-19 analytical sample (reported as unweighted N (adjusted percentages), except for age).

	No impairment (N = 4057)	Mild impairment (N = 1036)	Dementia (N = 114)	Overall (N = 5116)
Age (*mean (SE)*)	70.7 (0.16)	79.9 (0.31)	78.7 (0.98)	73.1 (0.16)
Female	2184 (53.0)	607 (58.6)	59 (57.0)	2850 (54.5)
Ethnic minority background	94 (3.2)	52 (6.3)	7 (11.3)	153 (4.2)
Lives with a partner/spouse	2862 (68.7)	564 (50.4)	69 (56.9)	3495 (64.0)
Education (high)	1060 (22.6)	77 (5.2)	16 (9.4)	1153 (18.0)
Education (medium)	1947 (46.8)	351 (26.6)	42 (30.8)	2340 (41.5)
Education (low)	975 (30.7)	596 (68.2)	52 (59.8)	1623 (40.5)
Employment status (in work)	736 (21.6)	44 (4.6)	4 (2.7)	784 (17.0)
Employment status (retired)	3083 (72.8)	941 (89.7)	98 (91.5)	4122 (77.4)
Employment status (other)	163 (5.6)	39 (5.7)	8 (6.2)	210 (5.6)
Net wealth (poorest third)	1174 (35.7)	460 (51.3)	53 (53.5)	1687 (39.9)
Net wealth (middle third)	1320 (30.8)	350 (30.8)	34 (29.2)	1704 (30.8)
Net wealth (richest third)	1488 (33.5)	214 (17.9)	23 (17.4)	1725 (29.3)
Rural residence	1226 (27.5)	260 (22.0)	32 (25.9)	1518 (26.2)
Region (The North)	1085 (28.3)	291 (30.5)	25 (26.5)	1401 (28.8)
Region (The Midlands)	852 (20.0)	220 (18.4)	26 (23.2)	1098 (19.7)
Region (London & East)	848 (22.5)	216 (23.3)	32 (29.2)	1096 (22.9)
Region (The South)	1197 (29.2)	297 (27.9)	27 (21.2)	1521 (28.7)
Self-rated health (excellent/very good)	1793 (42.3)	284 (25.6)	21 (23.1)	2098 (37.8)
Self-rated health (good)	1410 (34.9)	389 (36.4)	31 (25.0)	1830 (35.0)
Self-rated health (fair or poor)	779 (22.8)	351 (38.0)	58 (51.9)	1188 (27.3)
Presence of 2+ chronic conditions	871 (23.1)	353 (37.9)	63 (59.9)	1287 (27.6)

### Bivariate descriptive analyses

Before looking at the fully adjusted associations between cognitive function and our outcomes of interest, we report the bivariate trends (with 95% confidence intervals), adjusting only for sample design and weighting. These results are summarised in Table 2, including adjusted percentage estimates by cognitive function group with 95% confidence intervals and unweighted sample sizes for each outcome of interest.

**Table 2:** Percentage of people by cognitive function group reporting outcomes of interest (Adjusted for sample design, with unweighted sample sizes).

	No impairment (N = 4183)		Mild impairment (N = 1091)		Dementia (N = 123)	
	Adj. % N (Total N)	95% CI	Adj. % N (Total N)	95% CI	Adj. % N (Total N)	95% CI
Shielding in April 2020	91.8 3,727 (4,028)	[90.8,92.8]	92.3 956 (1,032)	[90.2,94.1]	96.7 109 (114)	[92.0,98.7]
Shielding in June/July 2020	79.9 3,249 (4,028)	[78.4,81.3]	82.5 849 (1,032)	[79.7,85.0]	93.7 105 (114)	[87.8,96.8]
Shielding in Nov/Dec 2020	74.6 2,986 (3,934)	[72.9,76.2]	79.9 764 (950)	[76.6,82.8]	81.5 75 (93)	[71.2,88.7]
Probable infection by June/July 2020	7.8 300 (4,028)	[6.9,8.9]	7.7 73 (1,032)	[5.9,9.9]	5.0 7 (114)	[2.3,10.5]
Disruption in access to community health services by June/July 2020	34.9 1,401 (4,026)	[33.2,36.7]	37.6 387 (1,030)	[34.3,41.0]	44.1 52 (114)	[33.5,55.3]
Disruption in access to community health services by Nov/Dec 2020 (among those present at Wave 1)	18.5 680 (3,810)	[17.1,20.1]	20.5 174 (896)	[17.4,24.0]	21.9 17 (84)	[13.4,33.9]
Disruption in access to community health services by Nov/Dec 2020 (among those reporting disruption at Wave 1)	28.6 366 (1,318)	[25.7,31.7]	29.8 98 (338)	[24.6,35.7]	33.7 11 (39)	[19.2,52.1]
Cancelled hospital operation or treatment by June/July	18.0 707 (4,028)	[16.6,19.4]	23.1 223 (1,032)	[20.1,26.4]	22.1 27 (114)	[15.1,31.3]
Cancelled hospital operation or treatment by Nov/Dec (all respondents)	11.7 441 (3,933)	[10.6,12.9]	16.3 132 (949)	[13.4,19.7]	10.4 10 (91)	[5.5,18.8]
Cancelled hospital operation or treatment by Nov/Dec (among those booked)	24.4 441 (1,846)	[22.2,26.8]	30.8 132 (491)	[25.8,36.5]	19.8 10 (51)	[10.8,33.4]

For shielding, the vast majority of people in all cognitive function groups were shielding at all of the three time points for which we have measures. Nearly 92% (90.8–92.8) of those with no impairment were shielding in April compared to 96.7% (92.0–98.7) of those with dementia. Rates of shielding did decline over time, and this decline appears faster for those with no impairment than for those with dementia; in Nov/Dec, 74.6% (72.9–76.2) of those with no impairment were shielding compared to 81.5% (71.2–88.7) of those with dementia. Estimates for those with mild impairment were between these groups and, in Nov/Dec, were significantly higher at 79.9% (76.6–82.8) than the proportion of those with no impairment.

Probable infection was not highly prevalent in our analytical sample, with only 380 out of 5,174 respondents classed as likely infected by June/July 2020. Estimates according to cognitive function suggest that a lower proportion of those with dementia were likely infected (5.0% (2.3–10.5)) than those with either no or mild impairment (7.8% (6.9–8.9) and 7.7% (5.9–9.9) respectively), but there is significant overlap in the 95% confidence intervals.

Because of the way the questions were framed at the two waves, we can look at the disruption in community health and care services according to different timeframes. We found a higher estimate of those with dementia experiencing disruption by June/July, with 44.1% (33.5–55.3) reporting this compared to 34.9% (33.2–36.7) of those with no impairment and 37.6% (34.3–41.0) of those with mild impairment. For those present at both waves, less disruption was reported between June/July and Nov/Dec, with around 1 in 5 people reporting this and little difference across cognitive function groups. However, there did appear to be a legacy effect for disruption; when looking at those who reported disruption by June/July, 29.0% (26.5–31.7) reported difficulty in access by Nov/Dec.

Regarding cancellations in hospital-based operations or treatments, a higher proportion of those with mild impairment noted this in June/July than those with no impairment: 23.1% (20.1–26.4) compared to 18.0% (16.6–19.4). The estimate for those with dementia was similar to the other two groups at 22.1% (15.1–31.3). Among responses in Nov/Dec, this difference remained when considering all respondents, as 16.3% (13.4–19.7) of those with mild impairment reported a cancellation compared to 11.7% (10.6–12.9) of those with no impairment; around 10.4% (5.5–18.8) of people with dementia reported cancellation. When excluding those in Nov/Dec who said they did not have a hospital-based service booked, the difference between those with mild impairment and no impairment weakens in statistical significance, with estimates of 30.8% (25.8–36.5) and 24.4% (22.2–26.8) respectively.

### Multivariate regression results

**Figure 1:**
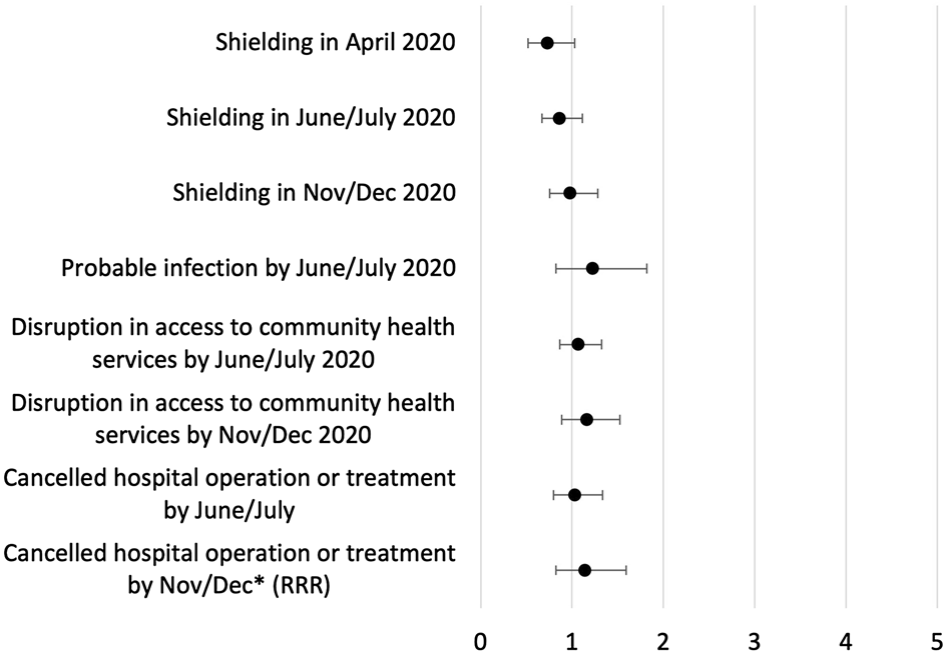
Differences between those with mild cognitive impairment compared to people with no impairment (multivariate regression). Results from logistic regressions, adjusting for demographics, socioeconomics, geography, and health. Odds Ratios reported except were indicated as RRR (Relative Risk Ratio). *RRR reported from multinomial logistic regression, comparing outcome to those not reporting a cancellation but with an operation/treatment booked.

**Figure 2:**
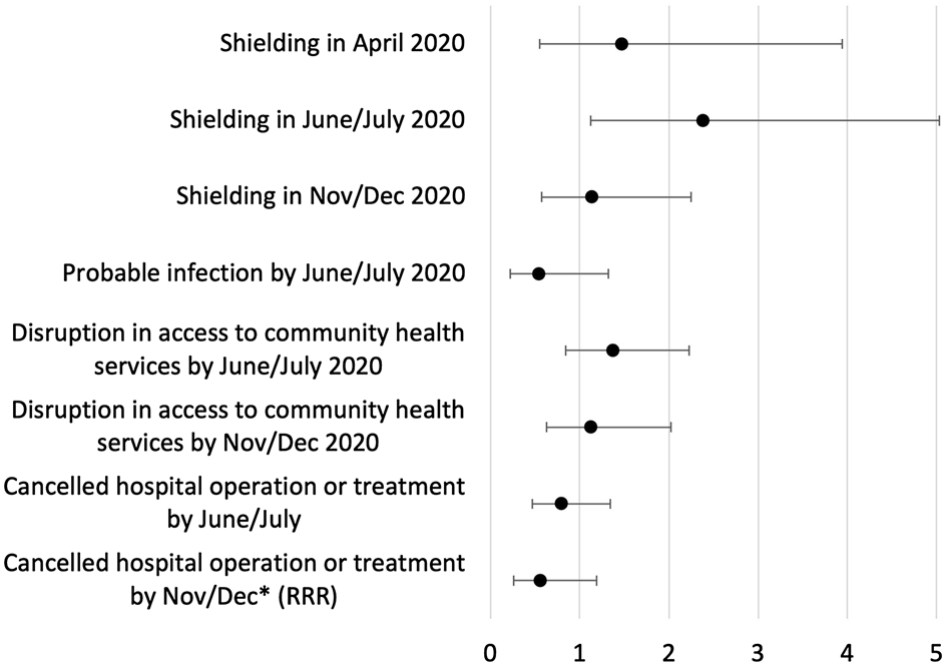
Differences between those with dementia compared to people with no impairment (multivariate regression). Results from logistic regressions, adjusting for demographics, socioeconomics, geography, and health. Odds Ratios reported except were indicated as RRR (Relative Risk Ratio). *RRR reported from multinomial logistic regression, comparing outcome to those not reporting a cancellation but with an operation/treatment booked.

Forest plots laying out the odds ratios (ORs) and 95% confidence intervals for the relationship between cognitive function groups and our outcomes of interest, adjusting for confounders, are featured in Figs. 1 and 2. The results for cancelled hospital-based services in Nov/Dec are relative risk ratios (RRRs), derived from multinomial logistic regressions and representing comparisons to the reference group of people who had such services booked but not cancelled.

Figure 1 captures the results for those with mild cognitive impairment compared to those with no impairment. There is weak evidence to suggest that those with mild impairment were around 27% less likely to be shielding in April 2020 compared to those with no impairment (OR = 0.729, 95%CI 0.517–1.029). With respect to disruption in access to community health and care services, the differences between people with mild impairment and those with no impairment were not statistically significant in either June/July or Nov/Dec 2020; it was a similar picture for hospital-based cancellations. No clear differences between those with mild impairment and those with no impairment were found related to probable infection with SARS-CoV-2 by June/July, shielding in Nov/Dec, or any outcomes in June/July.

Comparing people with dementia and those with no impairment (see Fig. 2), there was strong evidence to suggest they were 2.4 times more likely to be shielding in June/July (OR = 2.381, 95%CI 1.126–5.032). The other outcomes of interest showed no clear associations differentiating those with dementia from those with no impairment.

Overall, there was limited evidence of associations between cognitive function and our outcomes of interest. The only strong association was the higher likelihood of shielding in June/July among people with dementia compared to those with no impairment. Some covariates in our models, however, demonstrated consistent associations with our outcomes, highlighting their relative importance in shaping experiences of the pandemic; full regression results are available in Supplementary Tables S1–S3. Self-rated health was particularly important, with poorer health associated with negative experiences across all our outcomes of interest. The presence of multimorbidity was associated with worse outcomes related to disruption in access to service (Nov/Dec only) and hospital cancellations. For shielding, not being in paid work (classed as retired or other) as well as living with a partner were associated with higher likelihood of shielding for all three time points.

## Discussion

When controlling for a wide range of other factors, people with dementia were more likely than people with no impairment to be shielding in June/July, but there were no other strong associations between cognitive function status and shielding. Importantly, people with dementia were no more likely to experience disruption to services or hospital treatment during the pandemic. These results suggest that cognitive function status was not the primary driver of variations in the pandemic experience; poorer self-rated health proved to be a key predictor of negative experiences.

The strongest evidence we found was related to the higher likelihood of people with dementia to be shielding in June/July compared to those with no impairment. This may relate to the fact that society was just starting to reopen in the UK at this time, and people with dementia may have been more cautious than others. This could reflect a higher sensitivity to increased risk due to their cognitive status, thus greater desire to avoid such risk. On the other hand, it might reflect less confidence in their ability to follow guidelines or greater challenges in understanding what the guidelines mean in practice, as reported in earlier work^9–13^. In other words, they may have felt it was better to stay at home like in the previous months.

In our models for disruption in access, self-rated health demonstrated the only strong association with the likelihood of disruption in access at both time points, with those saying they had fair or poor health more than twice as likely as those with excellent or very good health to report disruption at either wave. Disruption in access may be linked to higher need stemming from overall health rather than from specific health conditions such as cognitive impairment. There is limited existing empirical research on the association between cognitive function and such disruption during the pandemic, though reduced contact with primary care among people with dementia was reported in one study^24^. Moreover, postponement of treatment during the pandemic was found outside the context of cognitive function, which may explain the greater impact of overall health and other factors in shaping engagement with care^17,18^.

Albeit not statistically significant, the estimates reflect patterns we would reasonably expect. Shielding was more prevalent with greater cognitive impairment, and the estimated odds ratios for those with dementia were greater than one compared to those with no impairment; we could expect people with dementia to shield at greater rates due to their increased risk.

Interestingly, results showed little difference between those with mild impairment and those with no impairment, including where differences for those with dementia were found. This may suggest that recognising cognitive impairment, such as with a formal diagnosis or well-understood symptoms apparent in more advanced impairment, plays a key role in stimulating adaptive health behaviours in a pandemic and beyond. Diagnoses can be required for people to access certain services, therefore greater effort and new strategies may be required to ensure diagnoses are made, including for those with mild forms of cognitive impairment.

Our study has several strengths. We used data on a representative sample of people aged 50+ in England, lending confidence that our findings apply to a broad population. The COVID-19 sub-study also achieved a notably high response rate for each wave of data collection and longitudinally. This strengthens our ability to generate robust results even during the public health crisis and social restrictions caused by the pandemic. Our study also incorporated a wide range of adjustment variables to control for confounding in our models, including linked data from ELSA waves collected before the pandemic.

A possible limitation of our study is that the relatively small number of people identified with dementia may influence our estimates’ significance and may reflect a lower likelihood that people with dementia responded to the survey despite high response rates overall. Moreover, a significant minority of people with dementia live in care homes (39%)^7^. Our findings are subsequently not applicable to the total population of people with dementia. Moreover, we must accept that varying levels of cognitive impairment could impact measurement error in terms of reliable recall. Nevertheless, the ELSA COVID-19 sub-study has proven to be an invaluable resource for understanding the impact of the pandemic among older people in the community, as it might be the best evidence to date.

To conclude, the likelihood of shielding and experiencing disruption in access to health and care services was no different according to cognitive function status when controlling for other factors like self-rated health. The only exception was that people with dementia had a higher likelihood of shielding early in the pandemic, although they were no more likely to experience disruption in access to community- or hospital-based services.

## Supplementary Information

Supplementary Tables.

## Data Availability

This study uses data from the English Longitudinal Study of Ageing, which are freely available upon registration through the UK Data Service.

## References

[1] Alzheimer’s Association (2019). 2019 Alzheimer’s disease facts and figures. Alzheimers Dement..

[2] Brown EE, Kumar S, Rajji TK, Pollock BG, Mulsant BH (2020). Anticipating and mitigating the impact of the COVID-19 pandemic on Alzheimer's disease and related dementias. Am. J. Geriatr. Psychiatry.

[3] Gerst-Emerson K, Jayawardhana J (2015). Loneliness as a public health issue: The impact of loneliness on health care utilization among older adults. Am. J. Public Health.

[4] Santini ZI (2020). Social disconnectedness, perceived isolation, and symptoms of depression and anxiety among older Americans (NSHAP): A longitudinal mediation analysis. Lancet Public Health.

[5] Steinman MA, Perry L, Perissinotto CM (2020). Meeting the care needs of older adults isolated at home during the COVID-19 pandemic. JAMA Intern. Med..

[6] Azarpazhooh MR (2020). Correlations between COVID-19 and burden of dementia: An ecological study and review of literature. J. Neurol. Sci..

[7] Alzheimer’s Society. Worst hit: dementia during coronavirus. (2020) https://www.alzheimers.org.uk/sites/default/files/2020-09/Worst-hit-Dementia-during-coronavirus-report.pdf (Accessed Apr 2022).

[8] Thyrian JR (2020). The situation of elderly with cognitive impairment living at home during lockdown in the Corona-pandemic in Germany. BMC Geriatr..

[9] Tsapanou A (2021). The impact of COVID-19 pandemic on people with mild cognitive impairment/dementia and on their caregivers. Int. J. Geriatr. Psychiatry.

[10] Hashimoto M (2020). The influence of the COVID-19 outbreak on the lifestyle of older patients with dementia or mild cognitive impairment who live alone. Front. Psychiatry.

[11] Kobayashi R (2020). Recognition of the coronavirus disease 2019 pandemic and face mask wearing in patients with Alzheimer's disease: An investigation at a medical centre for dementia in Japan. Psychogeriatrics.

[12] Suzuki M (2020). The behavioral pattern of patients with frontotemporal dementia during the COVID-19 pandemic. Int. Psychogeriatr..

[13] Tsugawa A (2020). Awareness of the COVID-19 outbreak and resultant depressive tendencies in patients with severe Alzheimer's disease. J. Alzheimers Dis..

[14] Atkins JL (2020). Preexisting comorbidities predicting COVID-19 and mortality in the UK Biobank Community Cohort. J. Gerontol. A Biol. Sci. Med. Sci..

[15] Pan AP (2021). SARS-CoV-2 susceptibility and COVID-19 mortality among older adults with cognitive impairment: Cross-sectional analysis from hospital records in a diverse US metropolitan area. Front. Neurol..

[16] Wang Q, Davis P, Gurney M, Xu R (2021). COVID-19 and dementia: Analyses of risk, disparity, and outcomes from electronic health records in the US. Alzheimers Dement..

[17] Holmes EA (2020). Multidisciplinary research priorities for the COVID-19 pandemic: A call for action for mental health science. Lancet Psychiatry.

[18] Webb E (2021). Providing health services effectively during the first wave of COVID-19: A cross-country comparison on planning services, managing cases, and maintaining essential services. Health Policy.

[19] Bauer K, Schwarzkopf L, Graessel E, Holle R (2014). A claims data-based comparison of comorbidity in individuals with and without dementia. BMC Geriatr..

[20] Zhou F (2020). Clinical course and risk factors for mortality of adult inpatients with COVID-19 in Wuhan, China: A retrospective cohort study. Lancet.

[21] Suárez-González, A. *et al*. The impact of the first UK Covid-19 lockdown on carers and people living with low prevalence dementia: results from the Rare Dementia Support survey. medRxiv 20248455 [Preprint] (2020) (Accessed Apr 2022) 10.1101/2020.12.18.20248455.

[22] van Maurik IS (2020). Psychosocial effects of corona measures on patients with dementia, mild cognitive impairment and subjective cognitive decline. Front. Psychiatry.

[23] Giebel C (2021). Impact of COVID-19 related social support service closures on people with dementia and unpaid carers: A qualitative study. Aging Ment. Health.

[24] Tuijt R (2021). Remote primary care consultations for people living with dementia during the COVID-19 pandemic: Experiences of people living with dementia and their carers. Br. J. Gen. Pract..

[25] Clare L (2022). Impact of COVID-19 on 'living well' with mild-to-moderate dementia in the community: Findings from the IDEAL cohort. J. Alzheimers Dis..

[26] Liu KY (2021). Dementia wellbeing and COVID-19: Review and expert consensus on current research and knowledge gaps. Int. J. Geriatr. Psychiatry.

[27] Banks, J. *et al*. English Longitudinal Study of Ageing: Waves 0-9, 1998-2019. [data collection]. 37th ed. UK Data Service. SN:5050, 10.5255/UKDA-SN-5050-24.

[28] Marmot, M. *et al*. English Longitudinal Study of Ageing COVID-19 Study, Waves 1-2, 2020. [data collection]. 3rd ed. UK Data Service. SN:8688, 10.5255/UKDA-SN-8688-3.

[29] Steptoe, A., Batty, D., Brayne, C., & Llewellyn, D. English Longitudinal Study of Ageing: Harmonised Cognitive Assessment Protocol, 2018. [data collection]. 2nd ed. UK Data Service. SN:8502, 10.5255/UKDA-SN-8502-2.

[30] Cadar, D., Abell, J., & Steptoe, A. Cognitive impairment and dementia in older English adults: risk factors and diagnostic algorithms. In *The Dynamics of Ageing: Evidence from the English Longitudinal Study of Ageing 2002–2019 (Wave 9)* (eds Banks, J. B. *et al.*) (Institute for Fiscal Studies, 2020) 62–92. https://ifs.org.uk/publications/15065 (Accessed Apr 2022).

