# Supplementary material for: Associations of cognitive impairment with self-isolation and access to health and care during the COVID-19 pandemic in England
Source: Sci Rep. 2023 Mar 28;13:5026. doi: 10.1038/s41598-023-31241-3 (PMC10043531; doi:10.1038/s41598-023-31241-3)
Supplement: Supplementary file 1 — Supplementary Tables. [file 41598_2023_31241_MOESM1_ESM.pdf]

Associations of cognitive impairment with self-isolation and access to health and care during the COVID-19 pandemic in England

*Brian Beach[1] (b.beach@ucl.ac.uk)

Nicholas Steel[2] (n.steel@uea.ac.uk)

Andrew Steptoe[3] (a.steptoe@ucl.ac.uk)

Paola Zaninotto[1] (p.zaninotto@ucl.ac.uk)

[1] UCL Research Department of Epidemiology & Public Health, University College London, London, United Kingdom

[2] Norwich Medical School, University of East Anglia, Norwich, United Kingdom

[3] UCL Research Department of Behavioural Science & Health, University College London, London, United Kingdom

Supplementary Table S1: Full model results, outcomes on shielding (Reporting Odds Ratios (OR) and 95% Confidence Intervals with statistically significant results in bold)

	**Shielding in April**		**Shielding in June/July**		**Shielding in Nov/Dec**	
	**OR**	**95%CI**	**OR**	**95%CI**	**OR**	**95%CI**
**Cognitive function** (ref: No impairment)						
Mild impairment	0.73	(0.52-1.03)	0.86	(0.67-1.11)	0.98	(0.75-1.28)
Dementia	1.48	(0.55-3.94)	**2.38**	**(1.13-5.03)**	1.14	(0.58-2.25)
**Female**	**1.33**	**(1.04-1.72)**	1.14	(0.97-1.36)	**1.32**	**(1.12-1.56)**
**Age**	1.01	(0.99-1.04)	1.02	(1.00-1.04)	1.01	(0.99-1.03)
**Non-white**	0.86	(0.40-1.86)	1.17	(0.67-2.04)	1.15	(0.68-1.93)
**Cohabiting with partner**	**1.32**	**(1.01-1.75)**	**1.41**	**(1.17-1.71)**	**1.36**	**(1.13-1.63)**
**Education** (ref: Low)						
High	**1.68**	**(1.15-2.45)**	**1.32**	**(1.03-1.68)**	**1.32**	**(1.05-1.67)**
Medium	1.05	(0.80-1.39)	1.13	(0.93-1.37)	1.20	(1.00-1.45)
**Wealth** (ref: Poorest tertile)						
Middle	0.94	(0.69-1.27)	0.99	(0.80-1.22)	0.98	(0.79-1.21)
Richest	1.17	(0.82-1.67)	1.03	(0.81-1.31)	0.98	(0.78-1.24)
**Work status** (ref: Employed)						
Retired	**3.90**	**(2.87-5.29)**	**2.60**	**(2.09-3.22)**	**2.70**	**(2.18-3.35)**
Other	**2.85**	**(1.31-6.20)**	**3.33**	**(1.88-5.88)**	**2.47**	**(1.48-4.12)**
**Rural residence** (vs Urban)	0.90	(0.69-1.18)	0.96	(0.80-1.15)	0.94	(0.78-1.13)
**Region** (ref: The North)						
The Midlands	0.91	(0.65-1.28)	1.06	(0.84-1.34)	0.95	(0.75-1.20)
London & East	1.18	(0.82-1.69)	0.91	(0.71-1.17)	0.88	(0.68-1.12)
The South	1.28	(0.91-1.81)	1.09	(0.87-1.37)	0.87	(0.69-1.09)
**Self-rated health** (ref: Excellent/Very Good)						
Good	0.85	(0.64-1.12)	0.99	(0.82-1.19)	**1.29**	**(1.07-1.54)**
Fair/Poor	**1.56**	**(1.02-2.40)**	**1.55**	**(1.20-2.01)**	**1.76**	**(1.38-2.25)**
**Multimorbidity** (2+ chronic conditions)	1.24	(0.89-1.71)	1.07	(0.86-1.32)	**1.41**	**(1.14-1.76)**

Supplementary Table S2: Full model results, outcomes on disruption in access to health and care services (Reporting Odds Ratios (OR) and 95% Confidence Intervals with statistically significant results in bold)

	**Disruption in access to care services (June/July)**		**Disruption in access to care services (Nov/Dec)**	
	**OR**	**95%CI**	**OR**	**95%CI**
**Cognitive function** (ref: No impairment)				
Mild impairment	1.07	(0.87-1.32)	1.16	(0.89-1.52)
Dementia	1.37	(0.85-2.23)	1.13	(0.63-2.02)
**Female**	1.06	(0.92-1.22)	0.91	(0.76-1.09)
**Age**	1.00	(0.98-1.01)	0.98	(0.97-1.00)
**Non-white**	0.84	(0.54-1.30)	0.80	(0.48-1.35)
**Cohabiting with partner**	0.92	(0.78-1.08)	0.91	(0.74-1.11)
**Education** (ref: Low)				
High	**1.23**	**(1.01-1.50)**	1.25	(0.97-1.61)
Medium	1.03	(0.88-1.21)	1.08	(0.88-1.33)
**Wealth** (ref: Poorest tertile)				
Middle	1.12	(0.94-1.34)	1.14	(0.91-1.41)
Richest	1.11	(0.91-1.36)	0.89	(0.69-1.15)
**Work status** (ref: Employed)				
Retired	1.00	(0.81-1.23)	1.05	(0.81-1.37)
Other	1.01	(0.65-1.56)	1.23	(0.75-2.02)
**Rural residence** (vs Urban)	1.04	(0.90-1.21)	1.10	(0.91-1.34)
**Region** (ref: The North)				
The Midlands	0.95	(0.78-1.16)	0.94	(0.74-1.20)
London & East	0.99	(0.81-1.23)	0.86	(0.66-1.11)
The South	0.88	(0.73-1.06)	0.74	(0.59-0.94)
**Self-rated health** (ref: Excellent/Very Good)				
Good	**1.67**	**(1.42-1.95)**	**1.39**	**(1.13-1.70)**
Fair/Poor	**2.55**	**(2.10-3.08)**	**2.00**	**(1.58-2.53)**
**Multimorbidity** (2+ chronic conditions)	0.93	(0.78-1.10)	**1.28**	**(1.04-1.57)**

Supplementary Table S3: Full model results, outcomes on hospital cancellations (Reporting Odds Ratios (OR) and 95% Confidence Intervals with statistically significant results in bold)

	Hospital cancellations by June/July		Hospital cancellations by Nov/Dec	
	**OR**	**95%CI**	**OR**	**95%CI**
**Cognitive function** (ref: No impairment)				
Mild impairment	1.03	(0.80-1.33)	1.14	(0.82-1.59)
Dementia	0.80	(0.47-1.35)	0.56	(0.26-1.19)
**Female**	1.12	(0.94-1.35)	0.95	(0.75-1.20)
**Age**	1.01	(0.99-1.02)	1.00	(0.98-1.03)
**Non-white**	0.96	(0.58-1.60)	0.97	(0.49-1.89)
**Cohabiting with partner**	1.02	(0.83-1.25)	0.82	(0.64-1.06)
**Education** (ref: Low)				
High	0.89	(0.69-1.15)	1.11	(0.80-1.55)
Medium	0.93	(0.76-1.13)	1.25	(0.96-1.62)
**Wealth** (ref: Poorest tertile)				
Middle	1.09	(0.88-1.36)	1.05	(0.78-1.40)
Richest	1.20	(0.93-1.56)	1.11	(0.79-1.57)
**Work status** (ref: Employed)				
Retired	1.04	(0.78-1.38)	1.27	(0.87-1.86)
Other	1.55	(0.94-2.56)	**2.05**	**(1.07-3.94)**
**Rural residence** (vs Urban)	1.07	(0.88-1.29)	1.08	(0.84-1.39)
**Region** (ref: The North)				
The Midlands	0.83	(0.65-1.07)	0.97	(0.70-1.36)
London & East	0.98	(0.76-1.27)	1.13	(0.81-1.57)
The South	0.86	(0.68-1.10)	1.02	(0.74-1.41)
**Self-rated health** (ref: Excellent/Very Good)				
Good	**1.71**	**(1.39-2.11)**	0.97	(0.72-1.30)
Fair/Poor	**2.84**	**(2.27-3.56)**	**1.86**	**(1.37-2.52)**
**Multimorbidity** (2+ chronic conditions)	**1.45**	**(1.20-1.76)**	**1.51**	**(1.19-1.92)**
